# Management of Melanoma in Advanced Age: A Comprehensive Literature Review of Tumor Biology, Systemic Therapies and Geriatric Challenges

**DOI:** 10.3390/cancers18183057

**Published:** 2026-09-21

**Authors:** Nerina Denaro, Nikolaos Papadopoulos, Dimitrios Stylianakis, Michele Ghidini, Emanuela Passoni, Cinzia Solinas, Ornella Garrone

**Affiliations:** 1 Oncologia Medica, Fondazione IRCCS Ca’ Granda Ospedale Maggiore Policlinico, 20122 Milan, Italy; nerina.denaro@policlinico.mi.it (N.D.); ornella.garrone@policlinico.mi.it (O.G.); 2 Division of Early Drug Development for Innovative Therapies, European Institute of Oncology, Scientific Institute for Research, Hospitalization and Healthcare (IRCCS), 20141 Milan, Italy; 3 Department of Oncology and Hemato-Oncology (DIPO), University of Milan, 20122 Milan, Italy; 4 Department of Social Medicine, School of Medicine, University of Crete, 70013 Heraklion, Greece; med4560@edu.med.uoc.gr; 5 Medical Oncology Division, ASST Sette Laghi, 21100 Varese, Italy; michele.ghidini@asst-settelaghi.it; 6 Department of Medicine and Technological Innovation (DiMIT), University of Insubria, 21100 Varese, Italy; 7 Dermatology Unit, Fondazione IRCCS Ca’ Granda Ospedale Maggiore Policlinico, 20122 Milan, Italy; emanuela.passoni@policlinico.mi.it; 8 Medical Oncology, AOU Cagliari, Policlinico di Monserrato, 09042 Monserrato, Italy; c.solinas@aoucagliari.it

**Keywords:** melanoma in the elderly, systemic melanoma therapy, safety, immune checkpoint inhibitors (ICIs), BRAF/MEK therapy, geriatric oncology

## Abstract

**Background:** Melanoma disproportionately affects older adults, who account for a growing share of cases as populations age. Its incidence rises with age, and the number of ultra-octogenarians (≥85 years) is expected to triple by 2050, particularly in high-incidence regions such as Australia, North America and Europe. Moreover, older adults frequently present with substantial comorbidities, making management complex and complicating treatment decisions. **Methods:** This comprehensive review summarizes evidence concerning the clinicopathological characteristics, treatment patterns, systemic therapy outcomes, and geriatric considerations relevant to patients aged 75 years or older with melanoma. PubMed/MEDLINE was searched from January 2000 to March 2026. Reference lists of eligible articles and relevant reviews were examined to identify additional studies. Priority was given to studies reporting age-stratified outcomes for patients aged at least 75 years. Because relatively few studies reported outcomes specifically for patients aged ≥75 years, evidence using thresholds of ≥65, ≥70, or ≥80 years was included when directly relevant. Age thresholds are reported for each study and should not be considered interchangeable. **Results:** Most evidence specific to patients aged ≥75 years derives from retrospective cohorts and registries. Compared with younger patients, elderly patients more often present with melanomas of the head, neck, hands and feet, a higher prevalence of the nodular subtype, greater Breslow thickness, higher mitotic rates and lower tumor-infiltrating lymphocyte density. Diagnosis is frequently delayed due to difficulties with self-examination, visual impairment, benign age-related skin changes and social factors. Despite presenting such a high-risk disease, older patients are less likely than their younger counterparts to receive immunotherapy or BRAF/MEK-targeted agents. This treatment gap may reflect comorbidities, altered pharmacokinetics, concerns about toxicity, under-representation in pivotal trials, and limited caregiver support. Available real-world data suggest that the efficacy of immune checkpoint inhibitors and targeted therapies is comparable to that in younger adults, although treatment selection, discontinuation, hospitalization, and dose modification differ across studies. **Conclusions:** Melanoma in older adults has distinct clinical and pathological patterns, but treatment evidence for patients aged ≥75 years remains limited and subjected to selection bias. Tailored, geriatric-informed treatment strategies integrating tumor biology, comorbidity, functional status and patient preferences are needed to optimize outcomes for patients aged 75 years and older.

## Simple Summary

This review examines melanoma with a particular focus on patients aged 75 years and older, with emphasis on tumor biology, treatment patterns, systemic therapy, and geriatric assessment. Older patients are more often associated with thicker, ulcerated, and nodular melanomas that frequently arise on the head, neck, hands, or feet. Despite this higher risk profile, they are less likely to receive surgery, immunotherapy, or BRAF/MEK-targeted therapy, partly because of frailty, comorbidities, polypharmacy, limited social support, and under-representation in clinical trials. Available real-world data indicate that immune checkpoint inhibitors and targeted therapies can provide meaningful benefit in older adults; however, treatment interruption, dose modification, and hospitalization may be more common. Management should therefore be guided not by chronological age alone, but by tumor characteristics, physiological reserve, functional status, cognition, comorbidity, nutrition, and patient preferences, ideally within a multidisciplinary, geriatric-informed framework.

## 1. Introduction

**Figure 1:**
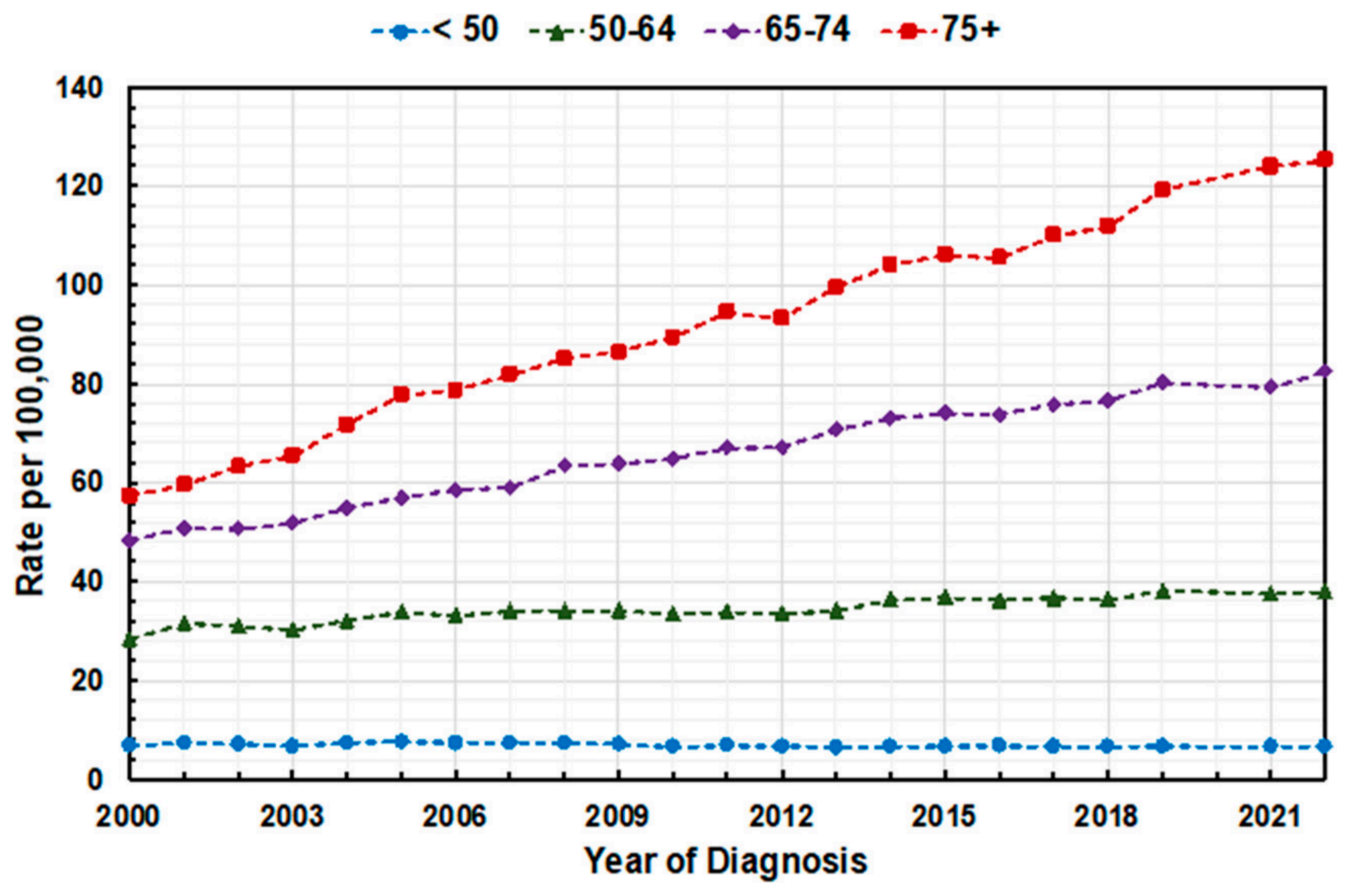
Age-adjusted melanoma incidence rates per 100,000 population in the United States (SEER Program), 2000–2022, stratified by age group. Dashed lines represent observed rates. <50 = Patients younger than 50 years of age; 50–64 = Patients aged between 50 and 65 years; 65–74 = Patients aged between 65 and 74 years; 75+ = Patients aged 75 years or older. Age-specific incidence of cutaneous melanoma in the United States, 2000–2022, according to age group. Data source: Surveillance, Epidemiology, and End Results Program. Age groups were <50, 50–64, 65–74, and ≥75 years.

Adults aged ≥75 years account for an increasing percentage of melanoma patients driven by population aging and the steep rise in incidence with advancing age [1,2]. The number of ultra-octogenarians (≥85 years) is projected to triple between 2020 and 2050. In high-incidence regions such as Australia, North America and Europe, melanoma rates in individuals aged 75 years and older exceed 120 cases per 100,000 person/years, far higher than in younger populations [1,2,3]. Recent data from the SEER program confirm this age-related gradient, with age-adjusted incidence rates of approximately 38 per 100,000 in people aged 50–64 years, 82.5 per 100,000 in those aged 65–74 years, and more than 120 per 100,000 in individuals aged ≥75 years (Figure 1).

Melanoma in older patients often presents distinctive clinicopathological features, such as thicker and more frequently ulcerated forms, lower lymphocytic infiltration and a preference for the head, neck or acral sites [4,5,6,7]. Despite this high-risk profile, elderly patients are less likely to receive systemic therapies such as immune checkpoint inhibitors or BRAF/MEK-targeted therapy. Concerns about toxicity in the context of comorbidities, polypharmacy, altered pharmacokinetics, reduced physiological reserve and limited social support, may be the origin of this selection bias [2,8,9,10]. Age-related immunosenescence may also modify antitumour immune responses and shift the risk–benefit balance of immunotherapy, creating a valid concern in the process of decision-making [11].

Previous reviews have addressed melanoma in older adults from epidemiological, biological, surgical and immunotherapeutic perspectives. Building on this literature the present review adopts a broader geriatric oncology perspective, integrating age-related tumor biology with surgical and systemic treatment, the representation of older adults in pivotal clinical trials, and the role of geriatric assessment in treatment decisions.

In this comprehensive literature review, we synthesize current data on the clinicopathological characteristics, management patterns and outcomes of melanoma in patients aged 75 years and older, examine the roles of frailty, comorbidity, and treatment selection. Our aim is to clarify how tumor biology and aging influence management, identify key evidence gaps, and provide practical, evidence informed guidance to support individualized treatment decisions for elderly patients with melanoma.

## 2. Materials and Methods

PubMed/MEDLINE was searched for English language articles published between January 2000 and March 2026 using the following MeSH and free text terms for melanoma and aging: (“melanoma”[MeSH] OR melanoma[tiab]) AND (“aged”[MeSH] OR elderly[tiab] OR older adults[tiab] OR senior[tiab] OR geriatric[tiab]) AND (“therapeutics”[MeSH] OR treatment[tiab] OR therapy[tiab] OR management[tiab]). Human studies reporting clinical, pathological, or treatment related data in older patients with melanoma were included. Studies were eligible if they enrolled adults with cutaneous melanoma and either included an identifiable cohort with a minimum age of 75 years or reported age-stratified outcomes that allowed extraction of data for patients aged ≥75 years. Because specific data was limited, studies using thresholds of ≥65, ≥70, or ≥80 years were included for contextual evidence and are identified as such. Non-English publications and geriatric oncology papers without melanoma-specific data were excluded.

Eligible designs included randomized and non-randomized trials, prospective and retrospective cohorts, registry analyses, case series, and systematic reviews; studies were grouped into themes covering epidemiology/clinicopathology, surgical management and sentinel lymph node biopsy, systemic therapy, and geriatric assessment and comorbidity. Given the heterogeneity in design and outcomes, no formal risk-of-bias assessment or meta-analysis was undertaken; instead, we synthesized findings qualitatively, highlighting data specific to patients aged ≥75 years.

## 3. Results

### 3.1. Overview and Determinants of Treatment in Elderly Patients

#### 3.1.1. Patient-Related Factors

Treatment decisions in older adults should be based on physiological reserve rather than chronological age alone. Patient-related considerations include polypharmacy, performance status, functional dependence in basic and instrumental activities of daily living, impaired mobility and risk of falls, swallowing difficulties, and nutritional status. The G8 screening tool is a tool that can help identify patients who may benefit from comprehensive geriatric assessment, whereas the Charlson Comorbidity Index estimates comorbidity burden. Neither tool, however, should be used in isolation to determine treatment eligibility. They could be used to determine physiological reserve, tolerance of anesthesia and systemic therapy, and the relative importance of survival versus quality-of-life endpoints in a shared decision-making multidisciplinary environment [8,12].

#### 3.1.2. Tumor-Related Factors

Tumor-related variables—such as stage at initial presentation, Breslow thickness and mitotic rate(although mitotic rate is no longer a primary AJCC T-category determinant, it may retain prognostic relevance), ulceration, surgical accessibility, and the presence or lack thereof of actionable mutations such as BRAF V600—directly influence the treatment decision. All these factors aid in deciding whether surgery, sentinel lymph node biopsy (SLNB), adjuvant systemic therapy or palliative options are appropriate [8,12,13]. High risk histological types, pathological stage and nodal status may justify SLNB and adjuvant therapy even in older patients, whereas thin lesions or indolent metastatic patterns may support less intensive strategies, particularly in the presence of substantial comorbidities.

#### 3.1.3. System and Center-Related Factors

In settings where geriatric assessment is not routinely available, frail older adults with melanoma are more likely to be undertreated, as clinicians may overestimate treatment related risks and underestimate potential benefits, which could lead to the use of less aggressive or nonstandard strategies [8]. Treatment choices may thus be driven by perceived frailty rather than by a systematic and objective evaluation of functional status, comorbidity, cognition and patient priorities. Integrating geriatricians into melanoma multidisciplinary teams—that typically include dermatologists, medical and radiation oncologists, surgeons and, in special cases, psychologists, nutritionists could eventually lead to more balanced, patient centered decisions and reduce cases of inappropriate undertreatment [6,13]. The current melanoma guidelines do not recommend treatment selection on the basis of chronological age alone. The appropriate surgical management, adjuvant or neoadjuvant treatment, systemic therapy for advanced disease should not be differential based on age. Although in patients of advanced age, the recommendations of international guidelines should also take into account functional status, comorbidities, polypharmacy, life expectancy and a geriatric assessment [14].

### 3.2. Clinicopathological Characteristics of Melanoma in Older Adults

#### 3.2.1. Anatomical Distribution and Histopathology

Older patients show a distinct anatomical distribution of primary melanoma, with a higher prevalence of lesions on the head and neck, hands and feet, and a relative decline in trunk and lower limb melanomas when compared with younger individuals [4,5]. The percentage of nodular melanoma increases with age and may be particularly high in cohorts of patients aged ≥80 years. Such tumor is typically thicker, more often with signs of ulceration, shows higher mitotic activity and harbors fewer tumor-infiltrating lymphocytes (TILS) [5,6,7]. The combination of aggressive histology and low lymphocytic infiltration is consistent with immunosenescence, which is the age-related decline in immune competence that may blunt anti-tumor responses [11].

#### 3.2.2. Age-Related Melanoma Biology

Beyond these differences, aging may influence melanoma progression through changes in both systemic immunity and the local tumor microenvironment. Immunosenescence, the natural, gradual decline of immune system function that happens with age is characterized by: thymic involution, a reduced T-cell receptor diversity and an altered antigen-presenting cell function. These changes and a chronic low-grade inflammation (“inflammaging”) may modify the fine balance between antitumour immunity and immune suppression. Another factor that can contribute to this process is the cellular senescence. Senescent immune cells remain metabolically active and continue to releasecytokines, chemokines, growth factors and matrix-remodeling mediators, a mechanism that could promote chronic inflammation. Moreover, oxidative stress and mitochondrial dysfunction also increase with age and may further contribute to genomic instability.

Aging is a factor that can also produce structural changes within the melanoma microenvironment that could directly influence tumor dissemination. In particular, dermal extracellular matrix has been shown to go through a phase of remodeling associated with age, in which it presents a reduced expression of hyaluronan and proteoglycan link protein 1 (HAPLN1) and an altered collagen architecture. Experimental data suggest that loss of HAPLN1 in aged tissues could provide an explanation, for the paradoxical observation that older patients can have lower rates of sentinel lymph node involvement despite poorer melanoma-specific outcomes [15,16].

#### 3.2.3. Molecular Profile and Nodal Involvement

Melanomas in elderly patients are frequently diagnosed at a more advanced stages and age-associated molecular differences may partly reflect cumulative sun damage. Older populations contain a larger proportion of NF1-mutated and triple-wild-type tumors, while BRAF V600 alterations are generally less frequent. Several studies have reported that BRAF mutations are less common in older than in younger patients, with BRAF-wild-type/NRAS-wild-type genotypes predominating in elderly cohorts, while BRAF-mutant/NRAS-wild-type profiles are more frequent in younger adults. Intermediate age groups show mixed patterns [8]. The BRAF-wild-type/NRAS-wild-type genotype may be associated with reduced nodal involvement in advanced stages, and paradoxically, elderly patients—despite having thicker, higher-stage primary tumors—often exhibit a lower incidence of sentinel lymph node metastasis [17,18].

The aging of the lymphatic system has been proposed as one explanation to this discordance between primary tumor risk and nodal status. Experimental and clinical data indicate that older adults have a diminished lymphatic flow and altered sentinel node uptake, which may favor haematogenous rather than a lymphatic dissemination [16,19,20]. In a large series of 4378 patients, Sasson et al. reported a higher proportion of T3–T4 and a lower proportion of T1 melanomas at initial presentation in older adults (*p* < 0.001), together with lower nodal positivity at any given Breslow thickness, and a rising prevalence of NRAS mutations with age [18]. Similarly, in ultra-octogenarians, Buja et al. described a markedly higher ulceration rate (43.3% versus 12.7%; *p* < 0.001), greater Breslow thickness (*p* < 0.001), fewer tumor-infiltrating lymphocytes (64.4% versus 76.5%; *p* < 0.01), and more advanced pTNM stage at diagnosis when compared with younger patients [5].

Beyond the BRAF, NRAS and NF1 alterations, emerging molecular markers may provide additional information on the metastatic behavior of the melanoma. Nicotinamide N-methyltransferase (NNMT) is a metabolic enzyme involved in nicotinamide methylation that through immunohistochemical studies has been shown to demonstrate differential expression between benign nevi, primary melanomas and lymph node metastases, a promising molecular marker that has yet to enter clinical practice [21,22].

#### 3.2.4. Diagnostic Delay and Stage at Presentation

Multiple factors contribute to delayed diagnosis in elderly individuals. Delayed detection may reflect lesions in difficult-to-examine sites, visual or mobility limitations, reduced opportunities for skin examination, and lower or infrequent access to dermatological care. Lesions being frequently located in difficult to inspect anatomical sites; age-related increases in benign lesions such as seborrhoeic keratoses that can mask melanoma and reduce vigilance; visual impairment, diminished attention to personal care and social factors such as widowhood or living alone further delay diagnosis [2,23,24,25]. Once a sentinel lymph node is positive, older patients are also less likely to undergo completion lymphadenectomy; in one specific study, rates of SLNB and lymphadenectomy in elderly versus younger patients were 60.0% versus 94.2% and 44.4% versus 85.5%, respectively (*p* < 0.001) [5].

### 3.3. Sentinel Lymph Node Biopsy

#### 3.3.1. Prognostic Value and Emerging Alternatives

The role of SLNB in elderly patients is increasingly debated. Dixon et al. applied prediction tools to model SLNB positivity and melanoma-specific mortality across ages and reported that SLNB has poor specificity for predicting mortality in young patients and poor sensitivity in older patients [26]. In their model, predicted SLNB positivity rates were much higher than mortality in 20-year-olds, whereas in the case of 80-year-olds the opposite was true, with many patients dying of melanoma despite negative sentinel nodes. Based on these findings, the authors argued that SLNB is not indicated for patients under 40 or over 60 years of age and proposed the BAUSSS clinical prediction model. This model is an algorithm incorporating Breslow thickness, age, ulceration, histological subtype, sex and site—a more appropriate tool according to them to guide adjuvant treatment decisions [26].

This modeling analysis generated a controversial age-based proposal that has not been incorporated into major clinical guidelines. Its conclusions depend on predicted rather than observed individual benefit and do not account fully for the contemporary staging, prognostic, and treatment-selection roles of SLNB. Nevertheless, the study highlights that mortality in elderly patients is driven by multiple factors beyond nodal status and highlights the need to consider both competing non-melanoma mortality and systemic treatment eligibility when interpreting SLNB results [17,26].

#### 3.3.2. Individualized Decision-Making in Older Adults

In day-to-day clinical practice, the indication for SLNB in older adults should be individualized, weighing the tumor thickness, ulceration and other high-risk tumor features against comorbidities, functional reserve, life expectancy and patient’s preference, ideally within a multidisciplinary framework. For some frail elderly patients, wide local excision alone may be appropriate, whereas for fit individuals with high-risk primaries, SLNB can still provide prognostic and staging information that may influence eligibility for adjuvant therapy. This nuanced approach aligns better with the heterogeneity of aging than using rigid age-based cut-offs.

**Figure 2:**
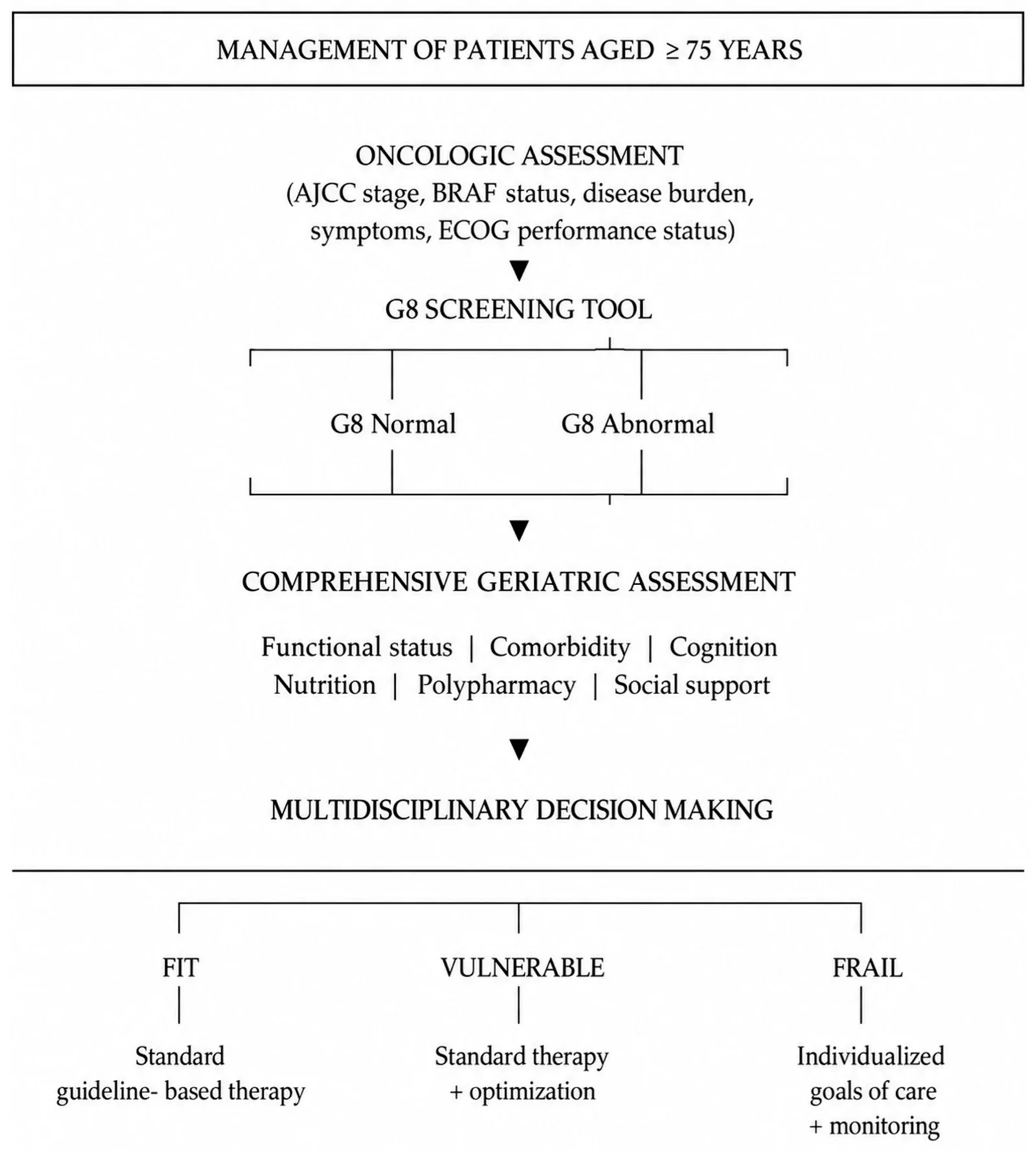
Proposed multidisciplinary framework for treatment selection in patients aged ≥75 years with melanoma. Initial treatment planning should integrate tumor characteristics with a geriatric assessment rather than relying on chronological age alone. Patients with an abnormal G8 screening result should undergo comprehensive geriatric assessment to evaluate frailty, comorbidities, functional status, cognition, nutrition, and social support. Treatment intensity should ultimately be individualized according to physiological reserve, expected benefit, and patient goals of care.

The factors discussed above should not be considered in isolation but should be integrated into a structured multidisciplinary decision-making process. Based on the available evidence and current geriatric oncology principles, Figure 2 proposes a pragmatic framework for treatment selection in patients aged ≥75 years with melanoma. The algorithm is intended to support, rather than replace, individualized clinical judgment.

### 3.4. Immunotherapy/ Target Therapy

#### 3.4.1. Representation in Trials and Efficacy

Across pivotal melanoma trials, patients aged 75 years and older remain markedly under-represented. Adjuvant and advanced-stage trials such as CheckMate 238, COLUMBUS, COMBI-D, COMBI-I and KEYNOTE-054 did not report dedicated data for participants ≥75 years (Table 1). Despite this under-representation and the well-established negative prognostic impact of older age [8,27], available data do not support excluding elderly patients from immunotherapy a priori.

Several studies examined both melanoma-specific and overall mortality, acknowledging that non-cancer deaths become more frequent with age. Older age may be prognostic for worse overall outcome without being predictive of reduced relative benefit from immunotherapy. Macdonald et al. observed worse five-year disease-specific and overall survival in patients older than 70 years, who also had a higher incidence of local and in-transit metastases [12]. However, analyses restricted to only treated patients suggest that immunotherapy efficacy is broadly preserved in older adults. In the pooled landmark analyses of the KEYNOTE 001, 002 and 006 trials, safety profiles were similar in patients aged ≥65 and <65 years [28]. A large meta-analysis of randomized trials including 17,476 patients reported identical hazard ratios for overall survival with immune checkpoint inhibitors in younger (<65 years) and elderly (≥65 years) groups (HR 0.77 in both), and no statistically significant age-related difference in progression-free survival. However, identical hazard ratios do not necessarily imply equivalent absolute benefit or comparable toxicity across age groups. [29]. Trial-level subgroup analyses generally do not demonstrate a statistically significant interaction between age and ICI efficacy. However, most analyses use a threshold of 65 years and include few adults aged ≥75 years, and in doing so provide only indirect evidence for the oldest patients. Table 1 summarizes the age-specific representation only when the relevant age subgroup was explicitly reported, or it could be directly calculated from data provided in the original trial publication or its supplementary material; otherwise, the corresponding field is reported as not available (N/R).

#### 3.4.2. Safety, Toxicity and Frailty

Real-world data further support the feasibility of ICIs in the elderly. De Glas et al. analyzed 2216 metastatic melanoma patients aged ≥65 years in the Dutch Melanoma Treatment Registry and found that grade ≥3 toxicity was not associated with age, comorbidities, or WHO performance status. However, patients aged ≥75 years discontinued treatment due to toxicity more frequently, leading to fewer treatment cycles. These results are subject to treatment-selection bias, since patients considered unsuitable for ICI were not represented among treated cohorts [30]. It should also be noted that response rates to anti-PD-1 therapy remained around 40% in both the 65–75 and ≥75-year groups, and melanoma-specific survival was not significantly influenced by age or comorbidity [30]. An Italian expanded-access program of ipilimumab 3 mg/kg in patients >70 years with pre-treated melanoma reported an immune-related disease control rate of 38%, median progression-free survival of 4.0 months and median overall survival of 8.9 months, with 1- and 2-year overall survival rates of 38% and 22%, respectively; treatment-related adverse events were generally manageable and resolved within a median of two weeks [31].

An Italian Melanoma Intergroup series of 174 patients treated with nivolumab or pembrolizumab (median age 79 years) showed an objective response in about one-third of patients and a disease control rate of 56.3%. The median overall survival was 17.2 months, and a severe (grade 3/4) toxicity occurred in only 11 patients, with no treatment-related deaths [32]. The toxicities, in particular, were effectively managed with corticosteroids without the need for additional immunosuppressive agents [32]. Likewise, a cohort from the Wilmot Cancer Institute found no significant differences in immune-related adverse events between patients younger and older than 70 years, even among those with ECOG performance status ≥2 or high Charlson comorbidity scores, and no irAE-related deaths were observed in a frail subgroup with ECOG 3 [33].

Although frailty may not increase the incidence of irAEs, it can increase their clinical consequences, which include hospitalization and treatment disruption. In a prospective study of 92 older melanoma patients receiving ICIs, Bruijnen et al. reported grade ≥ 3 irAEs in 20% of patients, with no significant difference in incidence between fit and frail individuals. However, frail patients required hospitalization for irAEs significantly more often and showed a trend toward longer hospital stays and more frequent immunosuppressant use or treatment discontinuation [34]. This observation correlates with the fact that impaired performance status, multimorbidity, and geriatric vulnerability may reduce the physiological reserve available to withstand an adverse event. Thus, even when the incidence of irAEs is not increased, their consequences may be greater in vulnerable patients. These could result in hospitalization, treatment interruption, prolonged recovery, or loss of functional independence.

#### 3.4.3. Adjuvant and Combination Immunotherapy in the Elderly

Adjuvant anti-PD-1 therapy in older adults has also been extensively evaluated. Gawaz et al. examined patients treated with adjuvant nivolumab or pembrolizumab between 2018 and 2022 and found that safety was comparable between older and younger groups, with grade ≥3 irAEs in 6.0% versus 10.0% of patients, respectively [35]. However, distant metastasis-free survival was shorter in the elderly cohort (9.0 versus 14.0 months; *p* = 0.007). The cause of the shorter DMFS is uncertain and may be attributed to baseline prognostic imbalance or residual confounding, reflecting both tumor biology and competing risks [35].

Regarding combination immunotherapy, in a French multicentre retrospective series, Reichert et al. described 60 melanoma patients aged ≥80 years treated with nivolumab plus ipilimumab. The immune-related adverse events occurred in 63% of patients, and grade ≥ 3 irAEs in 25%, notably lower than the 55% grade 3/4 rate reported in the pivotal combination trial. This is likely because most elderly patients received attenuated or non-standard dosing schedules [10,36,37,38]. These data illustrate the feasibility of combination therapy in selected very old patients despite the lack of an age-specific optimal dosing regimen.

#### 3.4.4. Real-World Treatment Patterns and Undertreatment

Several registry analyses have compared outcomes by age at the population level. Jochems et al. showed that patients aged >75 years were less frequently treated with systemic therapy than younger patients, reflecting persistent undertreatment, yet among treated patients, relapse-free survival did not differ substantially between age groups [39,40]. Van der Kooij et al. reported that younger patients had better performance status and more frequent BRAF mutations, whereas older adults more often had NRAS-mutant disease, but overall rates of grade 3/4 adverse events after first-line systemic therapy were similar across ages, with severe colitis being more common in older patients. The overall survival, however, favored younger individuals due to higher non-melanoma mortality in the elderly [41]. In a complementary analysis, van der Ziel et al. documented a marked increase in immunotherapy use over the last two decades across all age strata, with survival improvements in patients <65 and 65–75 years, but not in those >75 years—likely reflecting lower adoption of an immunotherapy strategy in the oldest group [42].

Data from Cybulska-Stopa et al. on 499 patients treated with nivolumab or pembrolizumab in three age groups (80–100, 65–79 and <65 years) did not identify statistically significant differences in overall, progression-free or melanoma-specific survival, nor in overall response or disease control rates, although the observational design and sample sizes limit conclusions about equivalence. Immune-related adverse event rates, including grade 3/4 events, were also comparable across groups, reinforcing the notion that age alone should not be used as an exclusion criterion for anti-PD-1 therapy [43]. Together with the meta-analysis by Kim et al., these findings support offering ICIs to appropriately selected elderly patients, with careful attention to frailty and comorbidities [29].

### 3.5. Targeted Therapies

#### 3.5.1. Dabrafenib–Trametinib in the ELDERLYMEL Study

BRAF/MEK-targeted combinations are standard options for patients with BRAF-mutant melanoma in both the adjuvant and metastatic settings, particularly when rapid tumor shrinkage is required, in symptomatic disease, or when immunotherapy is contraindicated. However, elderly patients were often under-represented in pivotal trials, and concerns persist regarding tolerability, especially given the risk of pyrexia and other systemic adverse events.

The ELDERLYMEL study, a multicentre, non-interventional real-world cohort, compared dabrafenib plus trametinib in elderly patients aged ≥75 years (*n* = 29) versus younger patients <75 years (*n* = 130) with advanced BRAF V600-mutant melanoma [44]. Overall, 159 patients were included, and adverse event rates and grades were similar between age groups, but the toxicity profile differed: pyrexia was significantly less frequent in patients aged ≥75 years (13.8% versus 42.3%; *p* = 0.005). Dose intensity of dabrafenib and trametinib was more often reduced in elderly patients (*p* = 0.018), yet the study did not detect significant age-related differences in effectiveness, although the small older cohort precluded a robust equivalence assessment between groups, and in multivariable analysis, female sex, and not age, was the only factor independently associated with a higher risk of grade ≥3 adverse events [44].

Table 2 summarizes key clinical series reporting systemic therapies in elderly melanoma patients, highlighting age strata, treatment regimens and main efficacy and safety outcomes.

#### 3.5.2. Other Real-World Series in Very Old Patients

A retrospective Finnish study by Kohtamäki et al. evaluated 34 consecutive patients aged >75 years (range 75–89) with metastatic melanoma treated with BRAF inhibitors with or without MEK inhibitors [46]. Adverse events were frequent, but were generally managed with interruption or dose modification. Because the study lacked a younger control group, age-related differences in tolerability cannot be determined. The overall response rate was 62%, including complete responses in 27% and partial responses in 35% of patients; median progression-free survival was 8 months (range 0–57) and median overall survival 15 months (range 0–71) [46].

Taken together, these data indicate that, in carefully selected elderly patients with BRAF-mutated melanoma, targeted therapies and ICIs can achieve response rates and disease control comparable to those in younger populations, albeit with specific toxicity patterns and a greater need for dose adjustments and close monitoring.

To translate all these considerations into routine clinical practice, Table 3 provides a concise step-by-step framework/checklist for the assessment and management of older patients with melanoma.

## 4. Conclusions

Melanoma in elderly patients is both common and challenging to manage. Older adults tend to present with more aggressive tumors and worse outcomes than younger people, but it is still unclear how much of it is due to later diagnosis and how much reflects different tumor biology.

Because of this complexity, treatment cannot be decided on age alone. It should consider the specific tumor characteristics (such as stage, thickness, and mutation status), but also the patient’s comorbidities, medications, functional status, cognition, and social support. The relatively low rate of positive sentinel lymph nodes in older patients, despite the high percentages of thick melanomas, suggests that metastatic pathways may differ in advanced age, and that nodal status should be interpreted cautiously when planning an adjuvant strategy.

Treatment goals should not be modified based on chronological age alone. In patients with substantial frailty and a limited life expectancy, disease control while preserving function and their quality of life may take precedence over maximal treatment intensity. This requires transparent conversations with patients and families about prognosis, expected benefits and side effects. A multidisciplinary, geriatric-informed approach is essential, and future research should focus on optimizing dosing, managing toxicity, and improving quality-of-life outcomes for patients aged 75 years and older.

## Figures and Tables

**Table 1:** Age distribution in selected benchmark melanoma trials.

Trial	Patients Aged ≥ 65 Years (%)	Patients Aged ≥ 75 Years (%)
CheckMate 067	39.2	N/R
COMBI-D	27.9	N/R
COMBI-i	27.8	N/R
CheckMate 238	25.8	N/R
CheckMate 76K	41.8	N/R
KEYNOTE-054	24.6 (24.6)	N/R
KEYNOTE-716	39.0 (38.7)	N/R
OPACIN	NR	N/R
SWOG 1801	46.0	N/R
PRADO	NR	N/R
COLUMBUS	29	N/R
NADINA	N/R	N/R
CA184-024	31.9	N/R
EORTC 18071	17.7	N/R
RELATIVITY-047	N/R	N/R

**Table 2:** Clinical series of elderly melanoma patients treated with systemic therapies.

Study	N	Stage	Age Groups	Treatment	Principal Findings in Elderly Patients
Reichert et al., 2023 [38]	60	IV	≥80 years	Nivolumab + ipilimumab	Any-grade irAEs occurred in 63% of patients, with grade 3–4 irAEs in 27%; toxicity was greater with the Ipi3/Nivo1 regimen than with Nivo1/Ipi3.
Gawaz et al., 2024 [35]	456	III–IV NED	≥75 vs. <75 years	Adjuvant nivolumab or pembrolizumab	Patients aged ≥75 years had shorter DMFS than younger patients (9.0 vs. 14.0 months; *p* = 0.007), despite a lower incidence of grade ≥3 adverse events (6.0% vs. 10.0%).
González Barrallo et al., 2022 [44]	159	IV	≥75 vs. <75 years	Dabrafenib + trametinib	Pyrexia was less frequent in patients aged ≥75 years than in younger patients (13.8% vs. 42.3%; *p* = 0.005). Grade ≥3 adverse events were more common in women, whereas treatment efficacy was unaffected by age.
Jochems et al., 2021 [39]	3054	IV	≤65, 66–74, ≥75 years	ICI or targeted therapy	Toxicity was not influenced by age. Three-year OS declined with increasing age (13.7% in patients aged ≥75 years vs. 26.7% in those aged ≤65 years; *p* < 0.001), whereas differences in melanoma-specific survival were modest (30.4% vs. 34.0%; *p* = 0.005).
Ozkan et al., 2024 [40]	285	III–IV NED	65–74 vs. ≥75 years	Adjuvant ICI	The incidence of grade ≥3 irAEs was similar between patients aged 65–74 and ≥75 years (15.5% vs. 13.9%; OR 0.88). The presence of ≥3 comorbidities, rather than chronological age, was associated with shorter recurrence-free survival.
de Glas et al., 2021 [30]	2216	IV	65–75 vs. ≥75 years	ICI	Patients aged ≥75 years discontinued treatment more frequently because of toxicity; however, response rates (~40%) and melanoma-specific survival were not significantly influenced by age after adjustment for comorbidity.
Jourdain et al., 2024 [13]	344	I–IV	≥80 vs. <80 years	Adjuvant and metastatic systemic Rx	Patients aged ≥80 years were less likely to receive adjuvant immune checkpoint inhibitors (82% vs. 97%; *p* < 0.001) and first-line systemic therapy for metastatic disease (87.5% vs. 96%; *p* = 0.001), whereas the use of targeted therapy and subsequent treatment lines did not differ by age.
Lallas K 2025 [45]	2150		≥75 vs. <75	All treatments	Patients ≥75 years received S, RT or ST less. Patients aged ≥75 years were less likely to undergo surgery, radiotherapy, or systemic therapy than younger patients (all *p* < 0.05). In the adjuvant setting, systemic treatment was administered to 25.8% of patients aged ≥75 years compared with 46.1% of those aged <75 years.

Abbreviations: ICI, immune checkpoint inhibitor; NED, no evidence of disease; DMFS, distant metastasis-free survival; RFS, relapse-free survival; OS, overall survival; MSS, melanoma-specific survival; irAE, immune-related adverse event.

**Table 3:** Checklist for the assessment and management of older patients with melanoma.

Step	Assessment	Practical Action
1. Define the setting	Stage (TNM), resectability, disease burden, symptoms; BRAF V600 and PDL1 status	Identify the guideline-based standard treatment options independent of chronological age
2. Assess general fitness	ECOG PS, major comorbidities, polypharmacy, organ function, recent hospitalizations	Identify factors that may affect treatment feasibility or toxicity
3. Screen for geriatric vulnerability	G8 screening	If abnormal, perform or request comprehensive geriatric assessment (CGA)
4. Characterize vulnerabilities	Function (ADL/IADL), mobility/falls, cognition, nutrition, psychological status, social support and comorbidity	Identify potentially reversible deficits and search for supportive interventions
5. Estimate treatment benefit versus competing risks	Expected melanoma benefit, physiological reserve, life expectancy, and competing mortality	Avoid both undertreatment based on age alone and avoid treatment whose burden is unlikely to provide meaningful benefit
6. Select treatment	Integrate oncologic indication with geriatric findings	Fit patients: standard guideline-based therapy; vulnerable patients: standard therapy when feasible with optimization/support; frail patients: individualized treatment intensity and goals
7. Discuss the decision with the patient and the caregiver	Expected benefit, toxicity, treatment burden, functional consequences, and patient priorities	Shared decision-making
8. Reassess during treatment	Toxicity, function, cognition, nutrition, falls, adherence, new medications, and caregiver burden	Intervene early and reconsider treatment intensity when health status changes

## Data Availability

All data supporting the findings of this review are derived from the published studies included and are available within the article. No individual patient data were accessed or analyzed.

## Abbreviations

The following abbreviations are used in this manuscript:

ADL	Activities of Daily Living
AE	Adverse event
BAUSSS	Breslow thickness, Age, Ulceration, Subtype, Sex, Site
BRAF	B-Raf proto-oncogene, serine/threonine kinase
DMFS	Distant metastasis-free survival
ECOG	Eastern Cooperative Oncology Group
G8	Geriatric 8
IADL	Instrumental Activities of Daily Living
ICI	Immune checkpoint inhibitor
irAE	immune-related adverse event
LDH	Lactate dehydrogenase
MEK	Mitogen-activated protein kinase kinase
MSS	Melanoma-specific survival
NED	No evidence of disease
NRAS	NRAS proto-oncogene, GTPase
OS	Overall survival
PFS	Progression-free survival
PS	Performance status
RFS	Relapse-free survival
SEER	Surveillance, Epidemiology, and End Results
SLN	Sentinel lymph node
SLNB	Sentinel lymph node biopsy
TIL	Tumor-infiltrating lymphocyte
TT	Targeted therapy
WHO	World Health Organization
